# Folding Free Energy Determination of an RNA Three-Way Junction Using Fluctuation Theorems

**DOI:** 10.3390/e24070895

**Published:** 2022-06-29

**Authors:** Jaime Aspas-Caceres, Marc Rico-Pasto, Isabel Pastor, Felix Ritort

**Affiliations:** 1 Small Biosystems Lab, Condensed Matter Physics Department, Universitat de Barcelona, Carrer de Martí i Franquès 1, 08028 Barcelona, Spain; jaime.aspas@protonmail.com (J.A.-C.); mricopasto@gmail.com (M.R.-P.); i.pastordelcampo@gmail.com (I.P.); 2 Institut de Nanociència i Nanotecnologia (IN2UB), Universitat de Barcelona, 08028 Barcelona, Spain

**Keywords:** Jarzynski equality, Crooks fluctuation theorem, Bennet’s acceptance ratio, RNA folding, single-molecule force spectroscopy

## Abstract

Nonequilibrium work relations and fluctuation theorems permit us to extract equilibrium information from nonequilibrium measurements. They find application in single-molecule pulling experiments where molecular free energies can be determined from irreversible work measurements by using unidirectional (e.g., Jarzynski’s equality) and bidirectional (e.g., Crooks fluctuation theorem and Bennet’s acceptance ratio (BAR)) methods. However, irreversibility and the finite number of pulls limit their applicability: the higher the dissipation, the larger the number of pulls necessary to estimate ΔG within a few $k_{B}T$. Here, we revisit pulling experiments on an RNA three-way junction (3WJ) that exhibits significant dissipation and work-distribution long tails upon mechanical unfolding. While bidirectional methods are more predictive, unidirectional methods are strongly biased. We also consider a cyclic protocol that combines the forward and reverse work values to increase the statistics of the measurements. For a fixed total experimental time, faster pulling rates permit us to efficiently sample rare events and reduce the bias, compensating for the increased dissipation. This analysis provides a more stringent test of the fluctuation theorem in the large irreversibility regime.

## 1. Introduction

Thermodynamic properties of nucleic acids and proteins are commonly studied in bulk assays; however, since the advent of single-molecule technologies, molecular thermodynamics can be determined with unprecedented detail. Techniques such as fluorescence resonant energy transfer (FRET) and force spectroscopy techniques, such as laser optical tweezers, permit us to monitor reactions one molecule at a time [1,2]. By exerting forces at the ends of a biopolymer, it is possible to monitor unfolding/folding reactions from the recorded changes in extension, allowing us to measure folding free energies [3,4,5] and binding energy of ligands to substrates [6,7,8].

Two different kinds of experiments are usually performed: hopping and pulling experiments. In hopping experiments, the unfolding/folding and binding/unbinding reactions are investigated under equilibrium conditions [9,10,11,12,13,14,15]. The main limitation of these experiments is the height of the kinetic barriers that molecules must cross, which, if too high, do not permit sampling of the different conformations in the experimentally accessible time [16,17]. Instead, in pulling experiments, the force is ramped at a given rate and the unfolding/folding and binding/unbinding reactions are followed under nonequilibrium conditions. In pulling experiments, the barrier height is modulated with time, rendering the folding energy landscape accessible even for molecules with high kinetic barriers, such as long RNAs or proteins [18,19,20,21,22].

In pulling experiments, the optical trap is moved back and forth to mechanically unzip the molecule while a force-trap position curve is recorded. Some molecules exhibit a large hysteresis between the unfolding and folding trajectories, indicative of a high kinetic barrier. A useful approach to estimate the folding free energy from nonequilibrium experiments is the Crooks fluctuation theorem (CFT) [23] and its corollary, the Jarzynski equality (JE) [24], which relate the work done under nonequilibrium conditions with the equilibrium folding free energy.

The CFT is a bidirectional method that uses the unfolding and folding trajectories to derive the folding free energy, ΔG. In contrast, the JE is unidirectional, meaning it only considers the unfolding or folding trajectories to estimate ΔG. Here, we investigate the limitations of the CFT and the JE using an RNA three-way junction (3WJ) as a model system [25]. The 3WJ is an interesting example of an RNA with high kinetic barriers to unfold and fold, which shows significant work dissipation in pulling experiments. Besides the CFT and the JE, $\Delta G^{0}$ has been estimated using Bennet’s acceptance ratio (BAR) to compare the different methods. BAR minimizes the statistical variance of the free-energy estimator from the CFT. We compare the three methods under different conditions to study their performance in determining folding free energies from irreversible work measurements.

## 2. Materials and Methods

### 2.1. Synthesis and Single-Molecule Experiments

**Figure 1:**
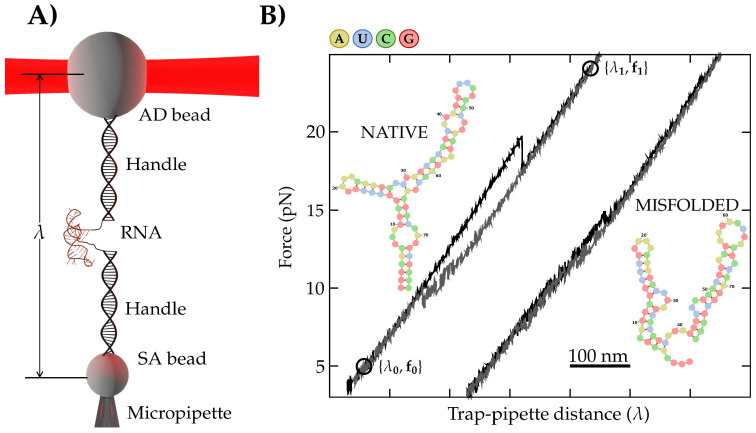
(**A**) Schematics of the experimental setup. λ stands for the trap-pipette distance, the control parameter in pulling experiments. (**B**) Force-distance curves (FDCs) (black, unfolding; grey, folding) and secondary structures (colored dotted-diagrams) of *native* (left) and *misfolded* (right) states in the RNA three-way junction (3WJ). FDCs have been horizontally shifted for clarity. Nucleotide color code shown on top.

The molecular construction has been synthesized following the protocol described in reference [3]. Briefly, the DNA sequence encoding the RNA 3WJ (Merck) is inserted between EcoRI (New England Biolabs, NEB, Ipswich, MA, USA) and HindIII (NEB) restriction sites of pBR322 vector (NEB) and cloned by ultra-competent *E. coli* cells (Invitrogen). Initially, T7 primers were used for PCR amplification (KOD polymerase, Merck, Township, NJ, USA) of the DNA containing the 3WJ sequence flanked by the handles. Next, RNA is synthesized by in-vitro transcription (T7 megascript, Merck). The transcribed RNA contains the 3WJ sequence flanked by 527 and 599 bases at the 3’-end and 5’-end, respectively. Hybrid DNA-RNA handles are made by hybridizing the RNA strand with PCR-amplified complementary DNA sequences. Handles are labeled with a biotin (5’-end) and a tail of digoxigenins (3’-end) that specifically bind to streptavidin-coated (SA) beads and anti-digoxigenin-coated (AD) beads. Schematics of the experimental setup are shown in Figure 1A. The molecular construction is tethered between the SA (2.1 μm Kisker Biotech) and AD beads (3.0–3.4 μm, Kisker Biotech). The SA bead is held by air suction at the tip of a glass micropipette, while the AD bead is captured in the optical trap.

Here, we have carried out non-equilibrium pulling experiments using a Mini-Tweezers optical setup [19] at room temperature (298 K) and monovalent salt conditions (300 mM NaCl) in a 10 mM HEPES buffer containing 1mM EDTA and 0.01% NaN${}_{3}$. The optical trap is moved up and down between two selected positions at a given pulling speed (Figure 1B). At the initial ($\lambda_{0}$) and final ($\lambda_{1}$) positions, the 3WJ is folded at a low force ($f_{0}$) and unfolded at a high force ($f_{1}$), respectively. Upon moving the optical trap up (down), unfolding (folding) events are observed as sudden force jumps in the force-distance curves (FDCs) (black and gray trajectories in (Figure 1B). The work, *W*, for the unfolding and folding trajectories is defined as the area below the FDCs between $\lambda_{0}$ and $\lambda_{1}$ [26].

### 2.2. Free-Energy Difference Determination: A Reminder

In this section, we summarize the main formulae for determining the free-energy difference between the folded and the unfolded RNA, ($\Delta G_{FU}$), from non-equilibrium pulling experiments. In the presence of a misfolded state, the standard Crooks fluctuation theorem (CFT) [23] and its corollary, the Jarzynski equality (JE) [24], are not directly applicable. Instead, one must use the extended versions of the CFT and the JE (hereafter referred to as ECFT and EJE) that consider the formation of different competing structures besides the native one [21,27,28]. In addition, we have also used the Bennet’s acceptance ratio (BAR) method [29] to extract ΔG. The different energy estimates are then compared to predictions computed using Mfold [30] and the Vienna package [31].

#### 2.2.1. The Extended Crooks Fluctuation Theorem (ECFT)

The CFT establishes a symmetry between the work distributions measured in a nonequilibrium process and its time-reverse, conditioned to start in full (Boltzmann–Gibbs) equilibrium at the beginning of each process. In the ECFT, this condition is replaced by the more general partial equilibrium condition; at the beginning of each process, the system is partially equilibrated in a given subset of the conformational space. In the pulling experiments shown in Figure 1, the RNA folds into two distinct conformations (subsets), native (*N*) and misfolded (*M*) [32,33]. Therefore, at the beginning of the pulling cycle, the RNA is partially equilibrated in *N* or *M*, and the ECFT must be applied. For simplicity, we will consider the *N* state, but the case for *M* is identical. Let $P_{\to }\left(W\right)$ and $P_{\leftarrow }\left(-W\right)$ denote the partial unfolding and folding work distributions conditioned to start in *N* at $\lambda_{0}$ and end in *U* at $\lambda_{1}$. Hereafter, the subscripts ${}_{⇄}$ indicate the unfolding (→) and folding (time-reverse, ←) processes. The extended Crooks fluctuation theorem (ECFT) for the trajectories that are in *N* at $\lambda_{0}$ reads [27]:

(1) $$\frac{\phi_{\to }}{\phi_{\leftarrow }}\frac{P_{\to }\left(W\right)}{P_{\leftarrow }\left(-W\right)}=\exp\left(\beta \left[W-\Delta G_{NU}\right]\right),$$

where $\beta =1/k_{B}T$, with $k_{B}$ being the Boltzmann constant and *T* the temperature. $\Delta G_{NU}$ is the equilibrium free energy difference between *N* and *U*. $\phi_{\to }$ ($\phi_{\leftarrow }$) are the fraction of trajectories that start at *N* (*U*) in $\lambda_{0}$ ($\lambda_{1}$) and end in *U* (*N*) at $\lambda_{1}$ ($\lambda_{0}$) during the unfolding (→) and folding (←) processes. Notice that in the standard CFT, $\phi_{\to }=\phi_{\leftarrow }=1$, because of the full equilibrium condition. For our RNA molecule, $\phi_{\to }=1$ (the RNA is always unfolded at $\left\{\lambda_{1},f_{1}\right\}$), whereas $\phi_{\leftarrow }<1$ because the molecule can fold into *M* (Figure 1B). The work value where both distributions cross, $P_{\to }\left(W^{★}\right)=P_{\leftarrow }\left(-W^{★}\right)$, will be denoted as $W^{★}$ and is given by $W^{★}=\Delta G_{NU}-C$, where $C=-k_{B}T\log\left(\phi_{\to }/\phi_{\leftarrow }\right)=k_{B}T\log\left(\phi_{\leftarrow }\right)$ is denoted as the ECFT correction. $\phi_{\leftarrow }$ is estimated by classifying unfolding and folding FDCs in two subsets, *N* and *M*, depending on which state the RNA folds into upon refolding. The classification is made on the basis of the FDC pattern (Figure 1B). Equation (1) also holds for the *M*-subset of trajectories. The crossing work value in Equation (1) defines the ECFT estimate for $\Delta G_{NU}$.

#### 2.2.2. The Extended Jarzynski Equality (EJE)

A corollary of the CFT is the JE. Its extended version reads,

(2) $$\Delta G_{NU_{⇄}}=-k_{B}T\;\log\left[{\langle e^{-\beta W}\rangle }_{⇄}\right]+C\;,$$

with $C=k_{B}T\log\left(\phi_{\leftarrow }\right)$, and $\Delta G_{NU_{⇄}}$ being the two energies estimates obtained by exponentially averaging (${\langle \cdots \rangle }_{⇄}$) over each set of $n_{U(F)}$ unfolding → (folding ←) trajectories, respectively. In practical cases, $n_{U(F)}$ is finite and the exponential average is biased for a finite number of experiments. The bias of the EJE is defined as,

(3) $$B_{n}^{⇄}=-k_{B}T\;\log\left[{\langle e^{-\beta W}\rangle }_{⇄}\right]-\Delta G_{NU}+C$$

where the averages ${\langle \cdots \rangle }_{⇄}$ are taken over different sets of *n* samples.

For FDCs presenting high hysteresis (Figure 1B), the unfolding $\Delta G_{NU_{\to }}$ and folding $\Delta G_{NU_{\leftarrow }}$ estimates differ. To overcome this, it is preferable to combine the unfolding and folding work measurements (e.g., using the ECTF Equation (1) and BAR; see next) to estimate $\Delta G_{NU}$.

#### 2.2.3. The Bennet’s Acceptance Ratio (BAR)

The Bennett’s acceptance ratio (BAR) method minimizes the statistical variance of the free-energy estimator given by the CFT. Bennet demonstrated that the function $\Phi \left(W\right)={\left(1+\left(n_{U}/n_{F}\right)\exp\left[\beta \left(W-\Delta G_{NU}\right)\right]\right)}^{-1}$ is the one that minimizes the statistical variance of the estimator ΔG[29]. Rearranging Equation (1) and multiplying it by Φ(W), we obtain:

$$\Phi \left(W\right)P_{\to }\left(W\right)=\Phi \left(W\right)P_{\leftarrow }\left(-W\right)\cdot \exp\left[\beta \left(W-\Delta G_{NU}+C\right)\right]$$

Integrating over W give us the expected values of Φ(W) over the work distributions:

$${\langle \Phi \left(W\right)\rangle }_{\to }={\langle \Phi \left(W\right)e^{\beta W}\rangle }_{\leftarrow }\cdot \exp\left[\beta \left(-\Delta G_{NU}+C\right)\right]$$

Taking logarithms and defining $u=\Delta G_{NU}-C$, and the functions $z_{F}\left(u\right)$ and $z_{U}\left(u\right)$:

(4a) $$\begin{array}{cc} \; & z_{U}\left(u\right)=\log\left[\frac{1}{n_{U}}\sum_{i=1}^{n_{U}}\left(\frac{e^{-\beta W_{i}^{\to }}}{1+\frac{n_{U}}{n_{F}}e^{-\beta (W_{i}^{\to }-u)}}\right)\right], \end{array}$$

(4b) $$\begin{array}{cc} \; & z_{F}\left(u\right)=\log\left[\frac{1}{n_{F}}\sum_{i=1}^{n_{F}}\left(\frac{1}{1+\frac{n_{U}}{n_{F}}e^{\beta (W_{i}^{\leftarrow }+u)}}\right)\right], \end{array}$$

with $W_{i}^{\to },W_{i}^{\leftarrow }$ being the work values measured in the unfolding and folding processes. In Equation (4), $n_{U}$ and $n_{F}$ denote the number of unfolding and folding trajectories, respectively. With these definitions, the ECFT takes the form:

(5) $$u=k_{B}T\;\left[z_{F}\left(u\right)-z_{U}\left(u\right)\right],$$

The solution to Equation (5), $u^{★}$, defines the BAR free energy estimate, $\Delta G_{NU}=u^{★}+C$.

#### 2.2.4. The Matching Method

An alternative and simple method to estimate $\Delta G_{NU}$, useful in those cases where work distributions do not cross, is the so-called matching method. In this method, we determine the value of $\Delta G_{NU}$ by imposing continuity between the measured $P_{\to }\left(W\right)$ and the reconstructed one from $P_{\leftarrow }\left(-W\right)$ using Equation (1):

(6) $$P_{\to }\left(W\right)=P_{\leftarrow }\left(-W\right)\cdot \exp\left(\beta \left[W-\Delta G_{NU}+C\right]\right)$$

The value of $\Delta G_{NU}$ that best matches the two work distributions in the crossing work region (around $W^{★}=\Delta G_{NU}-C$) defines the matching estimate [22]. Below, we will focus on the previous three estimates (ECFT, EJE, BAR), but will use Equation (6) to illustrate the matching method.

## 3. Results

**Figure A1:**
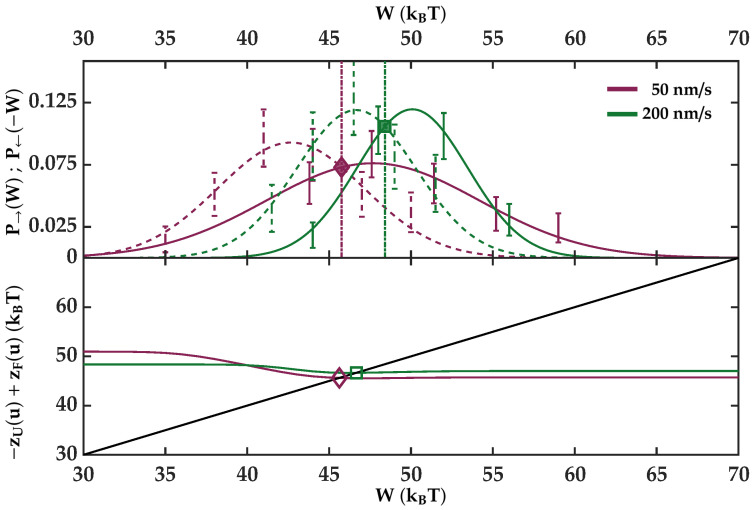
FT results for the misfolded structure. (**Top**) Partial work distributions for the misfolded structure. Solid (dashed) correspond to the unfolding (folding) work histograms with error bars computed by bootstrapping. Solid (dashed) lines are Gaussian fits to the data. The intersection between both distributions, $W^{★}$, is indicated with dotted vertical lines and by solid symbols. (**Bottom**) BAR method. The dark solid line represents the identity function and colored lines are the result for $z_{F}\left(u\right)-z_{U}\left(u\right)$. Empty symbols indicate $u^{★}$. In both panels, the diamond (square) symbols correspond to 50 (200) nm/s pulling speed.

We have carried out pulling experiments at two different pulling speeds: 50 nm/s and 200 nm/s. The recorded FDCs show two different patterns (Figure 1B) corresponding to two different folded structures: native and misfolded. We have determined the folding free-energy of the native state (*N*), $\Delta G_{N}$, using the three estimators described in Section 2.2 (ECFT, EJE, BAR). In the case of the misfolded state, as shown in Figure 1B, the trajectories for the misfolded state do not present a significant hysteresis, due to its quasi-reversibility in unzipping experiments. Therefore, we can just derive the free energy by taking the linear response formula to the first order, $\Delta G^{\left(1\right)}=\left({\langle W\rangle }_{\to }+{\langle W\rangle }_{\leftarrow }\right)/2$, or to the second order in a cumulant expansion, $\Delta G^{\left(2\right)}=\left({\langle W\rangle }_{\to }+{\langle W\rangle }_{\leftarrow }\right)/2-\left(\sigma_{\to }^{2}+\sigma_{\leftarrow }^{2}\right)/\left(12k_{B}T\right)$[34]. For the misfolded state, we found $\Delta G_{M}^{\left(1\right)}=48\left(1\right)k_{B}T$ and $\Delta G_{M}^{\left(2\right)}=48\left(1\right)k_{B}T$. From the ECFT and BAR, we obtain $\Delta G_{M}^{\left(\mathrm{ECFT}\right)}=51\left(1\right)k_{B}T$ and $\Delta G_{M}^{\left(\mathrm{BAR}\right)}=50\left(1\right)k_{B}T$. Averaging all estimators, we measured $\Delta G_{M}=49\left(1\right)k_{B}T$. All these results agree with the prediction using both Mfold and Vienna packages, which is $\Delta G_{M}^{0}=49\left(2\right)k_{B}T$ at our salt conditions. Results for the misfolded state can be found in Table A2 and are illustrated in Figure A1.

To determine $\Delta G_{NU}$ from the FDCs, we classify pulling trajectories into two sets (*N* and *M*), select the *N* subset, estimate the fraction of trajectories, $\phi_{\leftarrow }$, and measure the partial work distributions ($P_{\to }\left(W\right)$, $P_{\leftarrow }\left(-W\right)$). From the work *W*, we have subtracted the energy contributions coming from the bead displacement, the stretching of the DNA-RNA handles, and the 3WJ single-stranded RNA (ssRNA). To do so, we have followed the methodology described in [22,35]. Briefly, the elastic response of the ssRNA and hybrid DNA-RNA handles is calculated using the inextensible worm-like chain model [36]. The elastic parameters (*p* for persistence length and $d_{b}$ for the interphosphate distance) used in this study are: p=0.9 nm and $d_{b}=0.65$ nm/base for the ssRNA and p=40 nm and $d_{b}=0.3$ nm/basepair for the hybrid handles [35,37,38,39,40,41]. The folded hairpin is modeled as a freely-rotating dipole of 2 nm length [37]. The bead contribution has been estimated by solving the equation for λ(f) by applying the elastic models and using a harmonic optical trap with a cubic non-linear correction reported in previous experiments [35,42].

**Figure 2:**
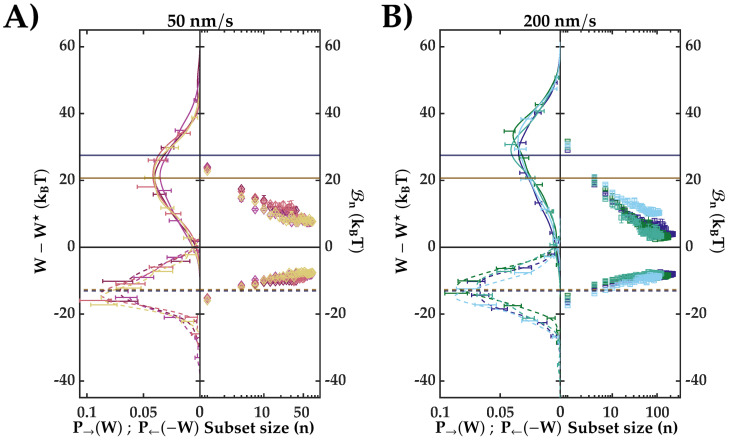
(**A**,**B**) **Left:** Partial work histograms for forward and reverse trajectories. Solid (dotted) lines are the generic kernel densities fitted to the forward (reverse) work measurements. Values for the histograms of $P_{\to }\left(W\right)$ and $P_{\leftarrow }\left(-W\right)$ are represented as error bars. These errors were computed by bootstrapping. (**A**,**B**) **Right:** Bias of the EJE (Equation 3) with the number of measurements. Errors have been computed with the variance of several random data subsets. In all panels, each color represents one of the eight different molecules. Horizontal lines indicates the mean value of the forward (solid lines) and reverse (dashed lines) distributions (color code: 50 nm/s dark yellow and 200 nm/s dark blue).

In Figure 2A,B (left) we show the unfolding (solid lines) and folding (dashed lines) work distributions for all investigated molecules at the two pulling speeds. From Equation (1), we derive $\Delta G_{NU}$ from the crossing work value. The crossing point has been estimated by fitting a generic kernel probability density distribution to the experimental histograms. In Figure 2, we have subtracted the crossing work value $W^{★}$ from the partial work *W*. We note that the unfolding work distributions are broader than the folding ones, suggesting that the transition state upon folding is closer to the native state. To characterize the work distributions, we have investigated their Gaussianity. To do so, we have defined a parameter *R* that relates the dissipated work, $\langle W^{\mathrm{dis}}\rangle =\left|\langle W\rangle -W^{★}\right|$, with the variance of the work, $\sigma_{W}^{2}$: $R=\sigma_{W}^{2}/\left(2k_{B}T\langle W^{\mathrm{dis}}\rangle \right)$; according to the ECFT, R=1 indicates perfect Gaussian behavior. For the folding work distributions, we find R=0.9(0.1) and R=1(0.1) for the 50 nm/s and 200 nm/s pulling rates, respectively. In contrast, the unfolding work distributions have R=2.0(0.2) and R=2.1(0.1) for 50 nm/s and 200 nm/s, respectively. This result indicates that the unfolding and folding work distributions have different behavior; only the folding distributions can be well approximated with a Gaussian function, which is suggestive of quasi-reversible folding. The average work values (〈W〉), variance (σ), and average dissipated work ($\langle W^{\mathrm{dis}}\rangle$) for the investigated loading rates are summarized in Table 1. The results for each studied molecule are gathered in Table A1 (Appendix A).

The biases, $B_{n}$, of the EJE estimator (Equation (3)) for different *n* values are shown in the right plots of Figure 2A,B. We have investigated data subsets of different sizes, each containing a random sample of work values. As expected, as the size of the subset grows, the bias decreases [43]. The free energy estimations using EJE Equation (2) are tabulated in Table 2. The average work values ${\langle W\rangle }_{\to }$ (${\langle W\rangle }_{\leftarrow }$) overestimate (underestimate) $W^{★}$ because the average dissipation ${\langle W^{\mathrm{dis}}\rangle }_{\to }={\langle W\rangle }_{\to }-W^{★}$ (${\langle W^{\mathrm{dis}}\rangle }_{\leftarrow }=W^{★}-{\langle W\rangle }_{\leftarrow }$) is always positive.

**Figure 3:**
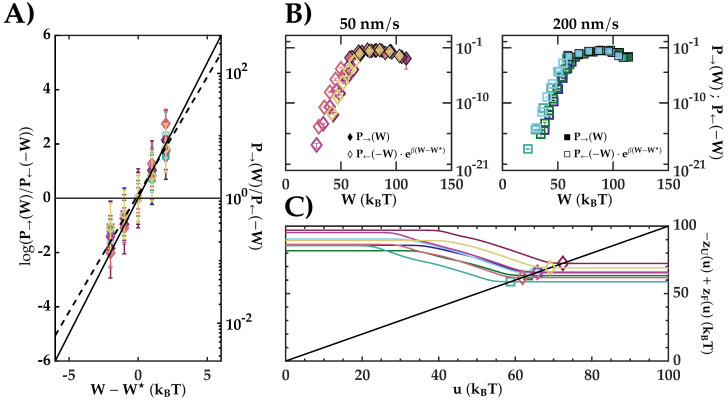
(**A**) Proof of the CFT applying Equation (1). The dark solid line represents the theoretical slope (1) and the dashed line is the result of a linear fit by considering all points. (**B**) Illustration of the matching method. Solid points are the direct histogram for $P_{\to }\left(W\right)$ and empty points are the reconstructed tails using Equation (6) (legend). (**C**) Result for the BAR. The dark solid line represents the identity function, while the colored lines are the result for $z_{U}\left(u\right)-z_{F}\left(u\right)$. Empty points are the intersection of both lines, $u^{★}$. For all panels, diamond (square) shaped points correspond to the pulling speed of 50 (200) nm/s.

As mentioned before, crossing work values ($W^{★}$) allow us to estimate the energy difference, $\Delta G_{NU}=W^{★}+C$. In Figure 3A, we tested ECFT Equation (1) using fits to the generic kernel densities by plotting the ratio between the unfolding and folding work distributions in a log-normal representation. As all energy values are presented in $k_{B}T$ units, the slope of Equation (1) is expected to be equal to 1 (dark solid line). The average slope (dashed line) equals 0.9±0.1, in agreement with the expected behavior (slope values for each molecule are shown in Table A1). In addition, we checked the ECFT by applying the matching method (Equation (6)). If the ECFT holds, we expect to see continuity between the measured (solid symbols) and the reconstructed $P_{\to }\left(W\right)$ using Equation (6) (empty symbols) (Figure 3B). Although there is continuity, we also appreciate a change in the slope around the crossing point. Moreover, we have estimated $\Delta G_{NU}$ using BAR (Figure 3C). The value of $u^{★}=\Delta G_{NU}-C$ is defined as the intersection (empty symbols) between the identity line (dark solid line) and the lines defined by Equation (5) (colored lines). As can be seen, the function $z_{F}\left(u\right)-z_{U}\left(u\right)$ is almost constant in a range of $∼4k_{B}T$ around the crossing point, $u^{★}$. Figure 3C shows the results using BAR for $\Delta G_{NU}$ for all molecules and pulling rates. Results are summarized in Table 2.

**Figure 4:**
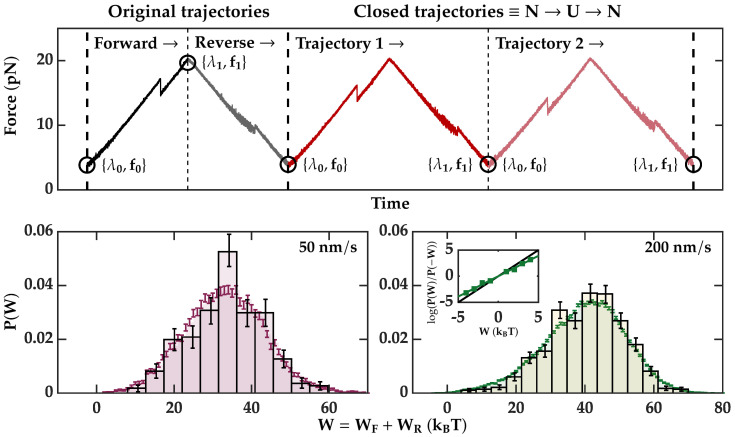
(**Top panel**). Scheme of the definition of the cyclic protocol. Dark (light) colors represent the forward (reverse) trajectories. (**Bottom**) Work distributions for the cyclic protocol (dark solid boxes) obtained by merging data from all molecules. Colored error bars correspond to the mixed data sets. Histogram errors are computed by bootstrapping. Left (right) panels are for pulling speeds of 50 (200) nm/s. (**Bottom, right, inset**) Proof of the CFT for the cyclic protocol. To test the CFT, we used the forward work distribution calculated by combining all unfolding and refolding trajectories. For the cyclic protocol, P(−W) is computed as the reflection of P(W) over the y axis. The green points were obtained by computing P(W)/P(−W) in the work range [−5:5] $k_{B}T$ and then fitting it to a line (green solid). The slope of the black solid line is equal to 1.

Finally, we tested the CFT over the pulling-cycle protocol defined by connecting the unfolding and folding processes, as shown in Figure 4. In this protocol, the trajectories are defined as the closed cycle determined by the unfolding and the subsequent folding trajectory. Therefore, the initial and final states are the same, i.e., the native molecule at $\left\{\lambda_{0},f_{0}\right\}$. By definition, in a cyclic protocol with a single control parameter, the forward and reverse work distributions are identical, i.e., $P_{\to }\left(W\right)=P_{\leftarrow }\left(-W\right)\equiv P\left(W\right)$ in Equation (1). The work for a single realization of a closed cycle is defined as $W=W_{U}+W_{F}$, with $W_{\left\{U,F\right\}}$ being the unfolding and folding work measurement values. If we restrict the analysis to native cycles (i.e., trajectories that start and end in N), the free energy difference for the closed cycle equals zero, $\Delta G_{NN}=0$ and the fractions $\phi_{\to }=\phi_{\leftarrow }<1$, so C cancels out and the ECFT Equation (1) takes the form:

(7) $$\frac{P(W)}{P(-W)}=\exp\left(\beta W\right),$$

In Figure 4 (bottom panels), we show the work histograms (dark solid boxes) for the cyclic protocol, P(W). Work histograms are shown for the two pulling speeds: 50 nm/s (bottom left), and 200 nm/s (bottom right). These histograms have been calculated considering all the trajectories from all molecules. Notice that the boxes do not reach W=0, and P(W) and P(−W) do not intersect at $W^{★}=0$, so we are not able to test the CFT as in Figure 3A. To circumvent this, we have combined the datasets of unfolding ($W_{U}$) and folding ($W_{F}$) work values. We have built a new dataset for the work cycle ($W=W_{U}+W_{F}$) by drawing $W_{U}$ and $W_{F}$ independently from each dataset. While the original histograms in Figure 4 are calculated with *N* closed trajectories, by mixing each unfolding trajectory with all the folding trajectories, we obtain a total of $N^{2}$ observations, which allow us to extend the tails of the histograms by increasing the number of trajectories from 457 (838) to 208,849 (702,244) at 50 (200) nm/s. The new histograms are displayed as error bars (red and green) in Figure 4, bottom. Note that with this procedure, we obtain virtual cycles that, despite never occurring, would be possible but extremely rare. As expected, the obtained distribution reasonably fits the original histogram. In exchange, we have obtained a few values for W<0 populating the leftmost tail of the histogram for the molecules pulled at 200 nm/s. These values allowed us to construct a P(W)/P(−W) and test Equation (1). The result is shown in the bottom right panel of Figure 4, inset. The dark solid line represents the unity slope. On the other hand, the same procedure over the dataset for the pulling velocity of 50 nm/s did not produce those values. Although a lower speed lowers the dissipation, this comes at the price of reducing the number of pulls for a given experimental time. Our results demonstrate that, for a given experimental time, it is preferably to increase dissipation by increasing the pulling speed and the number of cycles. In Table 3, we show the main parameters of the work distributions in the cyclic protocol.

## 4. Discussion

We have applied the Jarzynski equality (JE) and the Crooks fluctuation theorem (CFT) to determine the folding free energy of a native RNA three-way junction (3WJ), $\Delta G_{NU}$, from pulling experiments. The RNA 3WJ exhibits hysteresis between the unfolding and folding trajectories (see Figure 1B), which translates into a significant dissipation upon mechanical unfolding. The RNA 3WJ shows a misfolded two-hairpin structure [44] (Figure 1), which is predicted with the Vienna package [31]. Here we have focused on deriving the free energy of the native 3WJ only, where the crossing region between the unfolding and refolding work distributions ($P_{\to }\left(W\right)$, $P_{\leftarrow }\left(-W\right)$) is less populated and the determination of the folding free energy is more challenging.

In the presence of a misfolded structure, the standard JE and CFT must be corrected with the term $C=k_{B}T\log\left(\phi_{\leftarrow }\right)$, where $\phi_{\leftarrow }$ is the fraction of trajectories that fold into the native structure upon refolding. If there is no misfolding, $\phi_{\leftarrow }=1$ and C=0. We applied three estimators to the experimental data (Section 2.2): the extended Jarzynski equality (EJE), the extended Crooks fluctuation theorem (ECFT), and Bennet’s acceptance ratio (BAR). The average dissipated work for the unfolding process is ∼30 $\;k_{B}T$, which is almost half of the folding free energy (at zero force) predicted by Mfold [30,45]; $\Delta G^{0}=67\left(3\right)\;k_{B}T$ at [NaCl] = 1 M. This value must be corrected for salt dependence [37] to obtain a value of $\Delta G^{0}=62\left(3\right)\;k_{B}T$ in our experimental conditions. Combining the different estimators, we obtain $\Delta G^{0}=61\left(1\right)\;k_{B}T$ (see Table 2, bold), in agreement with the expected value.

We have applied the EJE to the unfolding and folding trajectories independently. In Figure 2 (right panels), we quantified the bias [46] of the EJE, B, and studied the convergence of the estimator with the number of pulls, following reference [43]. Despite the large average dissipation (∼20–30 $\;k_{B}T$ for unfolding and ∼$13\;k_{B}T$ for refolding), the EJE estimator converges after one hundred pulls. Averaging estimators for unfolding and refolding, we obtain $|\Delta G_{NU_{\to }}-\Delta G_{NU_{\leftarrow }}|$∼$12\;\left(8\right)\;k_{B}T$ for 50 (200) nm/s (see Table A1). We remark that faster pulling velocities allow us to obtain more pulls before the tether breaks, and a less biased energy estimator.

We have combined unfolding and refolding measurements in the ECFT (Equation (1)). This method yields a better estimate for the folding free energy (see Table 2). However, bidirectional estimators require that $P_{\to }\left(W\right)$ and $P_{\leftarrow }\left(-W\right)$ are sampled around the crossing region. A way to test the ECFT is shown in Figure 3, where a log-normal representation of Equation (1) shows a straight line with a slope close to 1 (see Table A1 for individual slopes). Further validation of Equation (1) is obtained with the matching method shown in Figure 3B, where continuity between the measured $P_{\to }\left(W\right)$ and the reconstructed one from $P_{\leftarrow }\left(W\right)$ using Equation (6) is imposed on the data to derive $\Delta G_{NU}$.

Bennett’s acceptance-ratio (BAR) method provides a third estimator of $\Delta G_{NU}$. In Figure 3C, we plot $z_{F}\left(u\right)-z_{U}\left(u\right)$ obtained by solving Equation (5). Although the difference is not flat throughout the u-axis, it is reasonably constant, with a range of $10\;k_{B}T$ around $u^{★}$, which give us confidence in the BAR results.

The RNA 3WJ was chosen because, despite its significant dissipation, there is a visible crossing point between unfolding and folding work distributions. This feature makes it an excellent candidate for comparing the different estimators.

Regarding the estimators, bidirectional methods (i.e., the extended Crooks fluctuation theorem (ECFT) and the Bennet acceptance ratio (BAR)) are more predictive because they combine data from unfolding and refolding in a unique formula. In contrast, the extended Jarzynski equality (EJE) uses the unfolding and folding work data separately. In general, when work distributions cross, it is advisable to take the crossing value as the free energy estimator. This includes the situation where dissipation is low (a few $k_{B}T$, for example, in the case of the misfolded structure). However, in this case, linear response theory provides good estimations for the free energy [34] (see Section 3, Results). If dissipation is large and the crossing work value is inaccessible, rare events must be sampled to reconstruct the leftmost (unfolding) and rightmost (folding) tails of the work distributions. In this case, it is advisable to increase the pulling speed to collect more trajectories for a given total experimental time (to more efficiently sample rare events) and apply the BAR method, which minimizes the statistical variance of the free energy estimator.

It is important to remark that, in the linear and low dissipation regime, work distributions tend to be Gaussian. In this case, the variance $\sigma_{W}^{2}$ and the mean dissipated work ($\langle W^{\mathrm{dis}}\rangle$) follow linear response theory, so $\sigma_{W}^{2}=2k_{B}T\langle W^{\mathrm{dis}}\rangle$. However, for dissipation comparable to $k_{B}T$, drift effects, finite measurement bandwidth, and experimental errors set a lower bound to the variance, so $\sigma_{W}^{2}>2k_{B}T\langle W^{\mathrm{dis}}\rangle$. Strictly speaking, Crooks FT does not hold; however, the crossing method and BAR still give good ΔG estimates. In particular, the Jarzynski equality overestimates the weight of the tails, yielding a negative free energy bias. This leads to misleading predictions; for instance, the unidirectional Jarzynski free energy estimator of the unfolding process is lower than the refolding one.

We want to remark that ΔG is an equilibrium quantity that does not depend on the pulling rate. Therefore, by collecting a sufficiently large number of trajectories, all the estimators would give the same ΔG value. However, as shown in Table 1, dissipation increases with the pulling rate, which would imply that more trajectories are necessary to estimate ΔG correctly. However, for a fixed total experimental time, faster pulling rates permit us to sample rare events much better, compensating for the increased dissipation.

Finally, we considered a new method of data analysis in which unfolding and folding trajectories are mixed to obtain full pulling cycles with ΔG=0. Forward and reverse work distributions are equal and the crossing equals $W^{★}=0$ by definition. The work histogram reaches $W^{★}=0$ for the largest pulling speed (Figure 4, 200 nm/s), and Equation (7) is fulfilled (inset). Future studies might address methods to increase the statistics around the crossing region to extract valuable information about the molecular folding energy landscape.

## Figures and Tables

**Table 1:** The subscripts ${}_{\to }$ and ${}_{\leftarrow }$ denote the unfolding and folding process. Energy values are expressed in $k_{B}T$ units. Values within parenthesis indicates the statistical error.

Pulling Speed	${\langle W\rangle }_{\to }$	$\sigma_{\to }$	${\langle W\rangle }_{\leftarrow }$	$\sigma_{\leftarrow }$	${\langle W^{\mathrm{dis}}\rangle }_{\to }$	${\langle W^{\mathrm{dis}}\rangle }_{\leftarrow }$	$R_{\to }$	$R_{\leftarrow }$
**50 nm/s**	82 (2)	8.8 (0.6)	49 (2)	4.9 (0.3)	20 (2)	13 (2)	2.0 (0.2)	0.9 (0.1)
**200 nm/s**	88 (2)	10.7 (0.2)	48 (2)	5.0 (0.2)	28 (2)	13 (2)	2.0 (0.2)	1 (0.1)

**Table 2:** Summary of the results for the different $\Delta G_{NU}$ estimators (see Table A1 for results for each molecule). EJE, ECFT, and BAR results are the mean value for all molecules at each pulling speed. $\Delta G^{0}$ is the mean value considering all the estimators. Energy values are expressed in $k_{B}T$ units. Values within parentheses indicates the statistical error.

	$\Delta G_{\mathrm{NU}}$				$\phi_{\leftarrow }$	C	$\Delta G^{0}$
	EJE→	EJE←	ECFT	BAR			
**50 nm/s**	65 (2)	58 (2)	59 (2)	65 (2)	0.53 (0.04)	−2.7 (0.4)	62 (2)
**200 nm/s**	62 (2)	57 (2)	60 (2)	62 (2)	0.77 (0.01)	−1.1 (0.1)	60 (1)
**Both**	63 (1)	58 (1)	59 (1)	63 (1)	-	-	**61 (1)**

**Table 3:** Characterization of the work distributions for the cyclic protocol. All energy values are expressed in $k_{B}T$ units.

	Data	〈W〉	$\sigma_{W}$	R
**50 nm/s**	Original	33	10	1.4
	Mixed	34	10	1.6
**200 nm/s**	Original	40	11	1.5
	Mixed	40	12	1.8

## Data Availability

The data presented in this study are available on request from the corresponding author.

## Appendix A. Results for the Native Structure

**Table A1:** Summary of the results for every native molecule. All energy values are expressed in $k_{B}T$ units.

Pulling Speed	50 nm/s				200 nm/s			
**Molecule**	**1**	**2**	**3**	**4**	**5**	**6**	**7**	**8**
**Total cycles**	73	172	91	121	297	231	166	144
**Native cycles**	39	71	52	73	227	181	125	114
${\langle W\rangle }_{\to }$	86	83	77	84	91	92	85	85
$\sigma_{{\langle W\rangle }_{\to }}$	9	10	8	9	11	11	11	10
${\langle W\rangle }_{\leftarrow }$	53	48	44	51	51	52	46	43
$\sigma_{{\langle W\rangle }_{\leftarrow }}$	5	6	5	5	5	5	6	5
${\langle W^{\mathrm{dis}}\rangle }_{\to }$	19	20	19	19	27	30	28	26
${\langle W^{\mathrm{dis}}\rangle }_{\leftarrow }$	14	14	14	14	13	10	12	17
**R** ${}_{\to }$	2.2	2.5	1.5	1.9	2.2	1.9	2.1	2
**R** ${}_{\leftarrow }$	0.74	1.2	0.8	0.8	1	1	1.3	0.7
${\mathrm{EJE}}_{\to }$	69	62	60	67	65	62	58	64
${\mathrm{EJE}}_{\leftarrow }$	61	60	56	58	61	59	54	53
**ECFT**	62	58	54	62	64	62	57	56
**slopes**	1.00	0.79	1.19	0.73	0.68	0.86	0.70	0.99
**BAR**	69	62	60	67	65	62	58	64
$\phi_{\leftarrow }$	0.5	0.4	0.6	0.6	0.8	0.8	0.8	0.8
C	−2.6	−3.6	−2.3	−2.1	−1.1	−1	−1.2	−1

## Appendix B. Results for the Misfolded Structure

**Table A2:** Summary of the results for every misfolded molecule. All energy values are expressed in $k_{B}T$ units.

Pulling Speed	50 nm/s				200 nm/s			
**Molecule**	**1**	**2**	**3**	**4**	**5**	**6**	**7**	**8**
${\langle W\rangle }_{\to }$	50	50	48	51	51	50	50	50
$\sigma_{{\langle W\rangle }_{\to }}$	5	3	4	5	3	4	3	3
${\langle W\rangle }_{\leftarrow }$	44	46	46	48	46	45	47	46
$\sigma_{{\langle W\rangle }_{\leftarrow }}$	5	4	4	4	4	4	4	4
${\langle W^{\mathrm{dis}}\rangle }_{\to }$	2	1	1	1	1	1	1	1
${\langle W^{\mathrm{dis}}\rangle }_{\leftarrow }$	3	3	2	2	4	4	4	4
$\Delta G^{\left(1\right)}$	47	48	47	50	49	48	49	48
$\Delta G^{\left(2\right)}$	47	48	47	49	49	48	49	48
**ECFT**	49	49	49	52	52	51	54	52
**BAR**	49	49	47	49	52	50	53	53
$\phi_{\leftarrow }$	0.5	0.6	0.4	0.4	0.2	0.2	0.2	0.2
C	−2.9	−2.1	−3.8	−3.8	−6.7	−6.7	−6.7	−6.7

## References

[1] Zaltron A., Merano M., Mistura G., Sada C., Seno F. (2020). Optical tweezers in single-molecule experiments. Eur. Phys. J. Plus.

[2] Bustamante C.J., Chemla Y.R., Liu S., Wang M.D. (2021). Optical tweezers in single-molecule biophysics. Nat. Rev. Methods Prim..

[3] Collin D., Ritort F., Jarzynski C., Smith S.B., Tinoco I., Bustamante C. (2005). Verification of the Crooks fluctuation theorem and recovery of RNA folding free energies. Nature.

[4] Woodside M.T., Block S.M. (2014). Reconstructing folding energy landscapes by single-molecule force spectroscopy. Annu. Rev. Biophys..

[5] Bustamante C., Alexander L., Maciuba K., Kaiser C.M. (2020). Single-molecule studies of protein folding with optical tweezers. Annu. Rev. Biochem..

[6] Rognoni L., Stigler J., Pelz B., Ylänne J., Rief M. (2012). Dynamic force sensing of filamin revealed in single-molecule experiments. Proc. Natl. Acad. Sci. USA.

[7] Kim E., Lee S., Jeon A., Choi J.M., Lee H.S., Hohng S., Kim H.S. (2013). A single-molecule dissection of ligand binding to a protein with intrinsic dynamics. Nat. Chem. Biol..

[8] Camunas-Soler J., Alemany A., Ritort F. (2017). Experimental measurement of binding energy, selectivity, and allostery using fluctuation theorems. Science.

[9] Woodside M.T., Behnke-Parks W.M., Larizadeh K., Travers K., Herschlag D., Block S.M. (2006). Nanomechanical measurements of the sequence-dependent folding landscapes of single nucleic acid hairpins. Proc. Natl. Acad. Sci. USA.

[10] Wen J.D., Manosas M., Li P.T., Smith S.B., Bustamante C., Ritort F., Tinoco I. (2007). Force Unfolding Kinetics of RNA Using Optical Tweezers. I. Effects of Experimental Variables on Measured Results. Biophys. J..

[11] Manosas M., Wen J.D., Li P., Smith S., Bustamante C., Tinoco I., Ritort F. (2007). Force Unfolding Kinetics of RNA using Optical Tweezers. II. Modeling Experiments. Biophys. J..

[12] Li P.T., Vieregg J., Tinoco I. (2008). How RNA Unfolds and Refolds. Annu. Rev. Biochem..

[13] Gebhardt J.C.M., Bornschlögl T., Rief M. (2010). Full distance-resolved folding energy landscape of one single protein molecule. Proc. Natl. Acad. Sci. USA.

[14] Elms P.J., Chodera J.D., Bustamante C.J., Marqusee S. (2012). Limitations of constant-force-feedback experiments. Biophys. J..

[15] Neupane K., Manuel A.P., Woodside M.T. (2016). Protein folding trajectories can be described quantitatively by one-dimensional diffusion over measured energy landscapes. Nat. Phys..

[16] Alonso-Caballero A., Tapia-Rojo R., Badilla C.L., Fernandez J.M. (2021). Magnetic tweezers meets AFM: Ultra-stable protein dynamics across the force spectrum. bioRxiv.

[17] Rico-Pasto M., Alemany A., Ritort F. (2022). Force-Dependent Folding Kinetics of Single Molecules with Multiple Intermediates and Pathways. J. Phys. Chem. Lett..

[18] Hummer G., Szabo A. (2010). Free energy profiles from single-molecule pulling experiments. Proc. Natl. Acad. Sci. USA.

[19] Huguet J.M., Bizarro C.V., Forns N., Smith S.B., Bustamante C., Ritort F. (2010). Single-molecule derivation of salt dependent base-pair free energies in DNA. Proc. Natl. Acad. Sci. USA.

[20] Edwards D.T., LeBlanc M.A., Perkins T.T. (2021). Modulation of a protein-folding landscape revealed by AFM-based force spectroscopy notwithstanding instrumental limitations. Proc. Natl. Acad. Sci. USA.

[21] Rissone P., Bizarro C.V., Ritort F. (2022). Stem–loop formation drives RNA folding in mechanical unzipping experiments. Proc. Natl. Acad. Sci. USA.

[22] Rico-Pasto M., Zaltron A., Davis S.J., Frutos S., Ritort F. (2022). Molten globule-like transition state of protein barnase measured with calorimetric force spectroscopy. Proc. Natl. Acad. Sci. USA.

[23] Crooks G.E. (1999). Entropy production fluctuation theorem and the nonequilibrium work relation for free energy differences. Phys. Rev. E.

[24] Jarzynski C. (1997). Nonequilibrium Equality for Free Energy Differences. Phys. Rev. Lett..

[25] Baker K.A., Lamichhane R., Lamichhane T., Rueda D., Cunningham P.R. (2016). Protein–RNA Dynamics in the Central Junction Control 30S Ribosome Assembly. J. Mol. Biol..

[26] Manosas M., Ritort F. (2005). Thermodynamic and kinetic aspects of RNA pulling experiments. Biophys. J..

[27] Junier I., Mossa A., Manosas M., Ritort F. (2009). Recovery of Free Energy Branches in Single Molecule Experiments. Phys. Rev. Lett..

[28] Alemany A., Mossa A., Junier I., Ritort F. (2012). Experimental free-energy measurements of kinetic molecular states using fluctuation theorems. Nat. Phys..

[29] Bennett C.H. (1976). Efficient estimation of free energy differences from Monte Carlo data. J. Comput. Phys..

[30] Zuker M. (2003). Mfold web server for nucleic acid folding and hybridization prediction. Nucleic Acids Res..

[31] Lorenz R., Bernhart S.H., Höner zu Siederdissen C., Tafer H., Flamm C., Stadler P.F., Hofacker I.L. (2011). ViennaRNA Package 2.0. Algorithms Mol. Biol..

[32] Li P.T.X., Bustamante C., Tinoco I. (2007). Real-time control of the energy landscape by force directs the folding of RNA molecules. Proc. Natl. Acad. Sci. USA.

[33] Zhong Z., Soh L.H., Lim M.H., Chen G. (2015). A U·U Pair-to-U·C Pair Mutation-Induced RNA Native Structure Destabilisation and Stretching-Force-Induced RNA Misfolding. ChemPlusChem.

[34] Hummer G. (2001). Fast-growth thermodynamic integration: Error and efficiency analysis. J. Chem. Phys..

[35] Severino A., Martinez-Monge A., Rissone P., Ritort F. (2019). Efficient methods for determining folding free energies in single-molecule pulling experiments. J. Stat. Mech. Theory Exp..

[36] Bouchiat C., Wang M.D., Allemand J., Strick T., Block S.M., Croquette V. (1999). Estimating the persistence length of a worm-like chain molecule from force-extension measurements. Biophys. J..

[37] Bizarro C.V., Alemany A., Ritort F. (2012). Non-specific binding of Na^+^ and Mg^2+^ to RNA determined by force spectroscopy methods. Nucleic Acids Res..

[38] Bosco A., Camunas-Soler J., Ritort F. (2013). Elastic properties and secondary structure formation of single-stranded DNA at monovalent and divalent salt conditions. Nucleic Acids Res..

[39] Yang D., Liu W., Deng X., Xie W., Chen H., Zhong Z., Ma J. (2020). GC-Content Dependence of Elastic and Overstretching Properties of DNA:RNA Hybrid Duplexes. Biophys. J..

[40] Zhang C., Fu H., Yang Y., Zhou E., Tan Z., You H., Zhang X. (2019). The Mechanical Properties of RNA-DNA Hybrid Duplex Stretched by Magnetic Tweezers. Biophys. J..

[41] Liu J.H., Xi K., Zhang X., Bao L., Zhang X., Tan Z.J. (2019). Structural Flexibility of DNA-RNA Hybrid Duplex: Stretching and Twist-Stretch Coupling. Biophys. J..

[42] Forns N., de Lorenzo S., Manosas M., Hayashi K., Huguet J.M., Ritort F. (2011). Improving signal/noise resolution in single-molecule experiments using molecular constructs with short handles. Biophys. J..

[43] Palassini M., Ritort F. (2011). Improving Free-Energy Estimates from Unidirectional Work Measurements: Theory and Experiment. Phys. Rev. Lett..

[44] Manosas M., Junier I., Ritort F. (2008). Force-induced misfolding in RNA. Phys. Rev. E.

[45] SantaLucia J. (1998). A unified view of polymer, dumbbell, and oligonucleotide DNA nearest-neighbor thermodynamics. Proc. Natl. Acad. Sci. USA.

[46] Gore J., Ritort F., Bustamante C. (2003). Bias and error in estimates of equilibrium free-energy differences from nonequilibrium measurements. Proc. Natl. Acad. Sci. USA.

